# Adherence to different methods for introducing complementary food to 7-month-old babies: a randomized clinical trial

**DOI:** 10.1590/1984-0462/2023/41/2021235

**Published:** 2022-09-09

**Authors:** Paula Ruffoni Moreira, Leandro Meirelles Nunes, Renata Oliveira Neves, Christy Hannah Sanini Belin, Jordana Fuhr, Erissandra Gomes, Adriela Mariath, Juliana Rombaldi Bernardi

**Affiliations:** a Universidade Federal do Rio Grande do Sul, Porto Alegre, RS, Brazil.

**Keywords:** Complementary food, Child nutrition, Clinical trial, Infant, Alimentação complementar, Alimentação infantil, Ensaio clínico, Lactente

## Abstract

**Objective:**

To assess the adherence to three methods of food introduction for 7-month-old babies.

**Methods:**

This is a randomized clinical trial conducted with mother-infant pairs, submitted to the intervention with five and a half months of age and three different methods for food introduction according to randomization: Parent-Led Weaning (PLW), Baby-Led Introduction to SolidS (BLISS), or mixed (specially developed for this study). Adherence to the method was assessed at the seventh month of age, via telephone call to the caregiver by a researcher blinded to the method. The analyses were performed using the Chi-Square test and data are presented in absolute numbers and percentages.

**Results:**

A total of 139 mother-infant pairs were evaluated; 46 of them were allocated to the PLW method; 47, to the BLISS; and 46, to the mixed. At seven months of age, 60 (43.2%) mothers reported that the infants were following the proposed feeding method. When analyzing each approach, the mixed method showed a higher likelihood of adherence (71.7%, n=33), followed by the PLW method (39.1%, n=18) and by the BLISS (19.2%, n=9) (p<0.001). Among the sample that did not follow the proposed method, those that had been randomized to the PLW and BLISS methods mostly migrated to the mixed method (92.9%; n=26 and 92.1%; n=35, respectively) (p<0.001).

**Conclusions:**

Complementary feeding in a mixed approach obtained greater adherence in 7-month-old babies.

## RESUMO

**Objetivo:**

Avaliar a adesão a três métodos de introdução alimentar aos 7 meses de vida.

**Métodos:**

Ensaio clínico randomizado com pares mães-lactentes submetidos à intervenção aos 5 meses e meio de vida sobre três diferentes métodos de introdução alimentar, conforme randomização: tradicional, Baby-Led Introduction to SolidS (introdução de sólidos guiada pelo bebê, em português) ou misto (criado especialmente para este estudo). A adesão ao método foi avaliada aos 7 meses em ligação telefônica feita para o cuidador por pesquisador cego em relação ao método. As análises foram realizadas por teste do qui-quadrado e os dados apresentados em número absoluto e percentual.

**Resultados:**

Foram avaliados 139 pares mães-lactentes, 46 alocados no método tradicional, 47 no *Baby-Led Introduction to SolidS* e 46 no misto. Aos 7 meses, 60 (43,2%) mães relataram que seus lactentes seguiam o método alimentar proposto. Analisando-se cada abordagem, o método misto apresentou maior probabilidade de adesão (71,7%, n=33), seguido do tradicional (39,1%, n=18) e de introdução de sólidos guiada pelo bebê (19,2%, n=9) (p<0,001). Da amostra que não seguiu o método proposto, aqueles que haviam sido randomizados para os métodos tradicional e de introdução de sólidos guiada pelo bebê migraram majoritariamente para o misto (92,9%; n=26 e 92,1%; n=35, respectivamente) (p<0,001).

**Conclusões:**

A alimentação complementar em abordagem mista obteve maior adesão aos 7 meses de idade.

## INTRODUCTION

The adequate introduction of complementary feeding (CF) is essential for the growth and development of infants.^1^ The Brazilian Ministry of Health recommends offering both soft foods in large pieces for the child to take to their mouths, and foods initially crushed with a fork or chopped, offered in a spoon, gradually progressing until reaching the consistency of the family's diet, at 12 months of age.^2^

In recent decades, alternative methods for introducing food, especially those led by the child, have been proposed such as the Baby-Led Weaning (BLW),^3^ and, later, the Baby-Led Introduction to SolidS (BLISS).^4^ Unlike the Parent-Led Weaning (PLW) method, in child-led techniques, the caregivers supervise, but do not take the food to the child's mouth, allowing the infant, from the onset of food introduction (FI), to eat the same meal consumed by the family, as long as it is in safe formats and consistencies.^5^

Many benefits are expected for children using alternative methods of CF^6^ such as the lower risk of consuming salt and sugar between 25 and 36 months of age,^7^ lower risk of high body mass index in infants fed with infant formula,^8^ greater exposure to consumption of vegetables and proteins,^9^ less agitation during meals^10^, and greater satiety responsiveness.^11^ However, the adherence of families to alternative methods of CF seems to be low, as demonstrated in a sample of children in Spain, where the prevalence of BLW, for example, was estimated at 2.1%.^12^ Confidence in the child and difficulty measuring the amount ingested are recurring concerns of mothers who adopt the BLW or BLISS methods to feed their children,^13,14^ which makes them choose for concomitantly using the FI method called mixed, in which they feed their children either with food cut into strips and sticks or with porridge and purees offered in a spoon.^14^

Despite the growing popularity of alternative FI methods among parents and healthcare professionals, and the increasing number of scientific publications on the topic, adherence to these methods is unknown in the Brazilian population. Thus, the objective of this study was to evaluate the adherence to three different methods of food introduction: PLW, BLISS, and mixed for 7-month-old babies.

## METHOD

**Figure 1:**
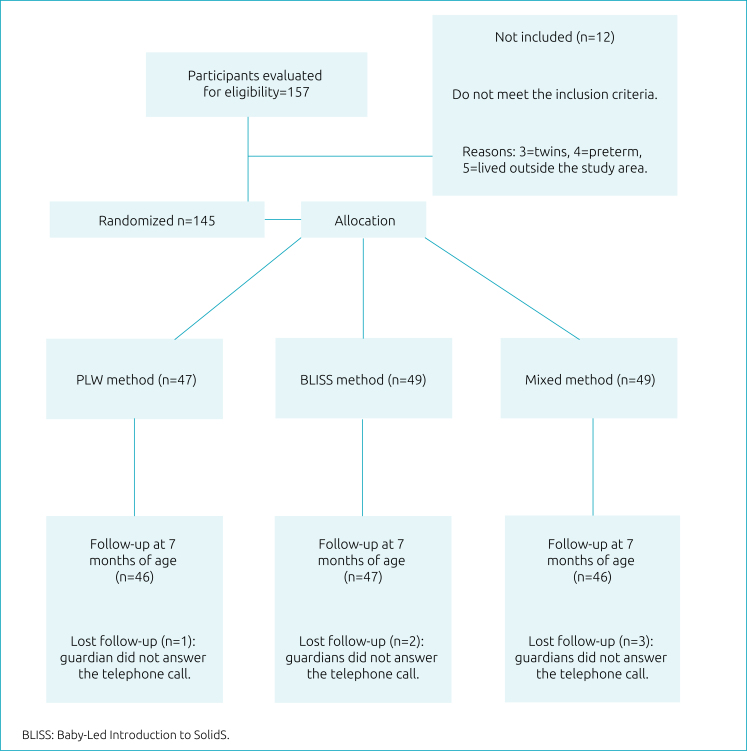
Study design.

Source: Prepared by the authors.

This is a three-arm randomized controlled clinical trial (Figure 1), involving different groups of mothers and infants about the method for introducing CF: PLW (A),^2^ BLISS (B)^4^, and mixed (C), the latter consisting of a combination between the PLW method and the BLISS, specially developed for this study.^15^

The project was approved by the Research Ethics Committee of Hospital de Clínicas de Porto Alegre under No. 2019-0230 and registered in the Brazilian Registry of Clinical Trials (*Registro Brasileiro de Ensaios Clínicos* – ReBEC) under identification RBR-229scm. The present study respects the bioethical aspects, according to Resolution No. 466 of December 12, 2012, of the National Health Council of the Brazilian Ministry of Health.^16^

Participants were recruited for the study via the Internet, both through social networks and websites and groups aimed at mothers, in addition to posters posted in opportune environments. The invitation letter contained a telephone number and an e-mail address that parents could contact and leave a message if they wished to participate. Once the inclusion criteria were verified, the subjects considered eligible received a standardized message explaining the details, risks, and benefits of the study, and participants’ doubts were answered by the researchers via telephone or e-mail. Upon confirmation of interest, the informed consent form was sent by e-mail.

After signing the online informed consent form, participants were randomized to one of the three intervention groups: PLW, BLISS, or mixed, through the website http://www.randomization.com, by a researcher blinded to the participants.

The following participants were considered eligible to participate in the research: mothers residing in the city of Porto Alegre (state of Rio Grande do Sul, Brazil) or in the metropolitan region, with full-term singleton newborns, with birth weight ≥2500g and who had not yet started food introduction. Only at the time of the intervention, the mothers were informed about the group to which they had been allocated.

When the babies aged five and a half months, the mothers were submitted to the intervention, which consisted of a food introduction workshop in a private nutrition clinic equipped with an experimental kitchen, in which previously trained nutritionists taught these mothers how to appropriately start FI, according to the group to which they were randomized. A speech therapist also provided guidance on choking prevention and management. The speech therapy team received prior training to standardize the guidelines to be provided. The workshop was offered to groups of four to seven mothers on a previously agreed date, according to the age of the infants. Participants and nutritionists prepared sample meals together, in real time, in the experimental kitchen.

Regardless of the CF method, the mothers were instructed to exclusively breastfeed for six months, and in a complementary way, for two years or more, in addition to being instructed on basic hygiene care in food preparation. Furthermore, they received support material specially produced for this study, according to the randomization group, as described next. Caregivers were trained and received printed material containing information on identifying signs of child's satiety.

Regarding the PLW method, the guardians were instructed to start offering solids from the sixth month of the child's life, with the slow and gradual introduction of other foods and offered in a spoon by the adult. The family should offer complementary foods (cereals, tubers, meats, legumes, and fruits) to the child three times a day, without rigid schedules and respecting their appetite, increasing this offer over the months; the consistency of the food should initially be pasty (from six to eight months, in the form of porridge and purees), and gradually progress until reaching the consistency of the family's food, at 12 months of age, with a variety of colors and food groups in all meals, without blending or sieving the food. Moreover, food preparations should be separated, in such a way that the infant assimilates the flavor and characteristics of each received food; it was recommended to avoid preparations with low energy density, such as soups and broths, in addition to sugar, coffee, canned goods, fried foods, soft drinks, juices, candies, snacks, and other sweets, in the first two years of life; salt should be used sparingly.^2^

Regarding the BLISS method, the guardians were instructed to encourage the infant to eat alone, although always assisted by an adult and participating in family mealtimes. The consistency of foods offered from six months of age onwards should be *in natura*, in formats that allow infants to feed themselves with their own hands, that is, cut into elongated formats, such as strips or sticks, which facilitate the movement of tweezers with the fingers and prevent choking, instead of rounded shapes. They were instructed to avoid rushing the child, respecting their time to explore flavors and textures while eating, offering them three types of food at each meal, which are sources of iron, for example, red meat; energy sources, such as tubers and cereals; and fibers, such as fruits or vegetables.^4^

As for the mixed method proposed by our research group, guardians were instructed to initially use the BLISS technique. If the child showed dissatisfaction or disinterest in food, according to the BLISS technique, they were instructed to offer the food using the PLW technique at the same meal.

During the intervention, the caregivers were instructed to contact the nutritionist responsible for the workshop to clarify any doubts about the provision of FI in the method to which they had been randomized, whenever deemed necessary.

At the infants’ seventh month of age, the mothers received a call from a participant of the research group, who was blinded to randomization, to verify adherence to the proposed method, based on a form containing keywords about each method, whether the infant took the food to the mouth and received it in a spoon most of the time.

The database was created using the Statistical Package for the Social Sciences (SPSS^®^), version 21.0, with double entry and subsequent validation. Data were presented in absolute numbers and percentages, with parametric values expressed as mean±standard deviation and nonparametric values as median and interquartile range. The Pearson's Chi-square or Fisher's exact tests were used to detect differences between proportions; and the Tukey's, Mann-Whitney, or Kruskal-Wallis *post hoc* ANOVA tests with Dunn's *post hoc* test were used to detect differences between means and medians. For all analyses, a significance level of 5% (p<0.05) and a confidence interval of 95% (95%CI) were considered.

The sample was estimated using the WinPepi^®^ software, considering a single standard deviation equal to 1, with a power of 80% and a significance level of 5%. The verified sample size calculation for a difference of half a standard deviation was 48 pairs of mothers-infants for each of the three intervention groups, totaling a sample of 144 pairs of mothers and their respective children, considering the performed studies.^4–10^

## RESULTS

The study flowchart is shown in Figure 1. For this study, the sample consisted of 139 mother-infant pairs. Of these, most women reported living with a partner (115; 82.7%), self-reported to be white (118; 84.9%), with a median (interquartile range) of 34 (30–36) years of age, and a monthly family income of BRL 6,000.00 (BRL 4,000.00–10,000.00). Regarding the type of delivery, 85 (61.2%) mothers reported they had a cesarean, most of them (112; 80.6%) primiparous.

After randomization of the sample to the methods for introducing complementary feeding, 46 (33.2%) pairs were allocated to the PLW method, 47 (33.8%) to the BLISS method, and 46 (33.2%) to the mixed method. Although the groups had similar medians in the age at which food was introduced, in the PLW group, the number of participants starting complementary feeding before the babies’ sixth month of age was significant compared with the BLISS group (p=0.046). The characteristics of the sample concerning the methods of food introduction are described in Table 1.

**Table 1:** Sociodemographic characteristics according to food introduction methods. Porto Alegre, RS.

		PLW (n=46)	BLISS (n=47)	Mixed (n=46)	p-value
Maternal age (years)*		33 (26–36)	35 (31–38)	33 (30–35)	0.082
Mother's level of education (years)*		17 (13–20)	18 (15–20)	18 (16–20)	0.491
Total family income (BRL)*		5,000 (3,925–10,000)	8,000 (4,000–13,500)	5,500 (3,875–10,000)	0.346
White maternal ethnicity		38 (84.4%)	40 (85.1%)	40 (87.0%)	0.952
Lives with a partner		36 (78.3%)	42 (89.4%)	37 (80.4%)	0.307
Primiparity		35 (76.1%)	37 (78.7%)	40 (87.0%)	0.400
Prenatal care consultations (number)		11 (10–12)	11 (10–12)	11 (10–13)	0.968
Cesarean delivery		33 (71.7%)	29 (61.7%)	23 (50.0%)	0.106
Child's sex					0.583
	Boy	19 (41.3%)	21 (44.7%)	24 (52.2%)	
	Girl	27 (58.7%)	26 (55.3%)	22 (47.8%)	
	Days of life for CF onset	180 (171–180)^A^	180 (180–180)^B^	180 (178–180)^AB^	0.046

Source: Prepared by the authors.

CF: complementary feeding; PLW: Parent-Led Weaning BLISS: Baby-Led Introduction to SolidS;

* variables that did not obtain the total n of the sample due to lack of data. Different superscript letters demonstrate statistically different results. Qualitative variables analyzed by Chi-square test, quantitative variables by Kruskal-Wallis with Dunn's *post hoc*, when nonparametric, and calculated by ANOVA with Tukey's *post hoc*, when parametric.

At the baby's seventh month of age, 60 (43.2%) mothers reported following the food supply according to the feeding method proposed in the intervention. By analyzing each approach, there was a higher probability of adherence to the mixed method for 7-month-old babies (71.7%, n=33), followed by the PLW method (39.1%, n=18) and the BLISS method (19.2%, n=9) (p<0.001). There was no statistically significant difference in adherence regarding the sex of the infants (p=0.770).

**Figure 2:**
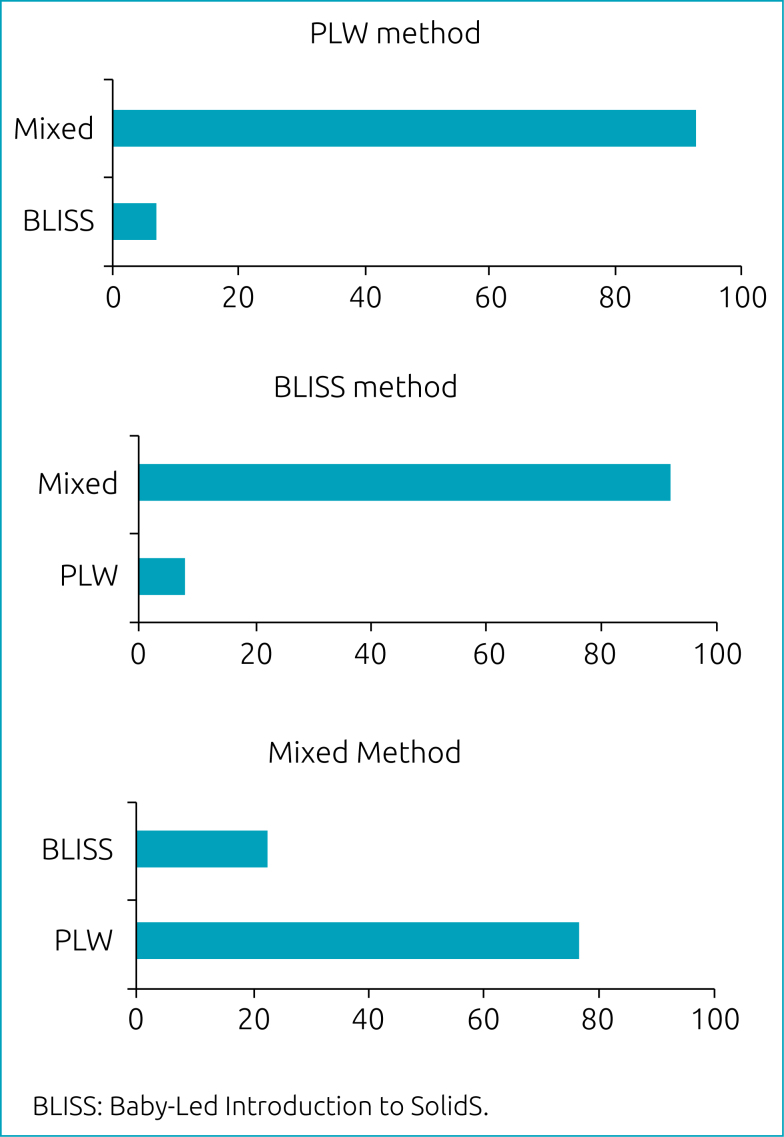
Migration among those who did not follow the proposed method with 7-month-old babies per randomization group. Porto Alegre, RS.

Source: Prepared by the authors.

Among the sample that did not follow the proposed method, those who had been allocated to the PLW and BLISS methods mostly adopted the mixed method (92.9%; n=26 and 92.1%; n=35, respectively), which received new members the most (77.2%; n=61). Likewise, 16.5% (n=10) of those allocated to the mixed method migrated to the PLW method (p<0.001) (Table 2 and Figure 2).

**Table 2:** Migrations between methods of food introduction for 7-month-old babies. Porto Alegre, RS.

		PLW	BLISS	Mixed	Total	p-value*
Method designated by randomization	PLW	0	2 (7.1)	26 (92.9)	28 (60.9)	—
	BLISS	3 (7.9)	0	35 (92.1)	38 (80.8)	—
	Mixed	10 (16.5)	3 (23.1)	0	13 (28.3)	—
	Total	13 (16.5)	5 (6.3)	61 (77.2)	79 (100.0)	0.001

Source: Prepared by the authors.

PLW: Parent-Led Weaning; BLISS: Baby-Led Introduction to SolidS;

* Chi-square test.

In Figure 2, the migration among participants who did not adhere to the method proposed in the intervention is described.

## DISCUSSION

The food introduction method specially developed for this study (mixed), which includes the offer of mashed or pureed foods in spoons and as strips or sticks, showed greater adherence by participants at the babies’ seventh month of age.

There was a statistical difference in the age of introduction of complementary feeding between the groups. Although the median age in days was the same, the interquartile range showed that a greater number of infants started complementary feeding before 180 days of life in the PLW group compared with the BLISS group. Similar results were found in other studies, such as the one conducted by Taylor et al., who found a significant difference between spoon-fed and BLISS-fed children in the duration of breastfeeding (median of 21.7 weeks; 95%CI 13.0–23.8 *versus* median of 17.3 weeks; 95%CI 6.0–21.7, p=0.002) and in the introduction of solids from six months of age onwards (64.6% in the BLISS-fed *versus* 18.1% in spoon-fed children, p<0.001)^10^; and the study performed by Morison et al., who demonstrated the later onset of the introduction of solids for children fed with the alternative method (21.3±2.8 weeks *versus* 24.6±2.0 weeks, p<0.001) and longer duration of breastfeeding (14.4±8.6 weeks in spoon-fed children *versus* 22.2±7.6 child-led alternative method, p=0.003).^17^ Conversely, in the research developed by Dogan et al., the introduction of complementary feeding in the child-led food introduction group took place approximately one week after the PLW group.^18^ The later onset of the introduction of solids, from the baby's sixth month of life onwards and based on signs of readiness present at the time, implies a longer period of exclusive breastfeeding, guaranteeing these children the benefits of breast milk.^19^

The adherence to the BLISS method was the lowest one verified in the present sample. As verified by a population-based study, this is the most infrequently adopted method by parents (18% in a sample of 876 children aged 6 to 36 months).^20^ Other studies have discussed the possible causes of low adherence to infant-led methods. A study carried out on 36 mothers of infants aged between 12 and 18 months showed that, although the experience of infant-led food introduction is positive, food waste and mess are frequent concerns^21^ and can impair the continuity of the method. The perception of little control over the amount of food ingested by the child reported by caregivers who follow the infant-led methods is also seen as a negative aspect for adherence to the method.^22^

Although alternative child-led methods have existed for more than two decades, mothers’ knowledge of them is limited. A Spanish study, whose authors evaluated 6,355 women aged between 18 and 50 years, showed that less than 40% of the participants had heard about the child-led methods, and of those who followed the method, 3.6%, with 4- and 5-month-old babies, and 3.2%, with 13- and 14-month-old babies, were more likely to breastfeed for longer, lived in urban areas, were under 40 years of age, and had a higher level of education.^12^ Thus, low adherence to the BLISS method may be related to maternal factors, as this requires the ability to understand and respond to the child's hunger and satiety signs, making responsive mothers better suited to the method.^23^

The style of parental care is also an important factor for adherence to the food introduction method, as it is suggested that following infant-led methods is associated with a low-control maternal feeding style.^24^ In addition to the style of parental care, the personality, feeding behavior, and well-being characteristics are significantly different between mothers who follow the PLW approach and those who adopt the infant-led approach.^25^ Willingness to follow recommendations on food introduction can also be a limiting factor for adherence, considering that the mothers claimed to do what is best for their child and do not follow the guidance of the healthcare professional.^26^

Healthcare professionals usually have limited knowledge of infant-led methods, in such a way that they may be reluctant to indicate them.^27^ Therefore, in the present study, participants received support to maintain the method, in addition to being instructed to communicate the child's participation in the study to their reference professionals, considering that lack of support from healthcare professionals can generate insecurity in the mother to maintain the recommended method of food introduction.^14^

Of the mothers who did not follow the BLISS method, most migrated to the mixed group. This behavior corroborates the findings of another study, in which mothers reported mixing the offer of food cut into sticks and strips with food offered in a spoon and in other textures to avoid mess and help their children when they were not able to feed themselves.^14^

It is noteworthy that the strengths of the study are the novelty in the area of complementary feeding, as the researchers proposed a new method of food introduction, called mixed, and nutritional intervention, providing a dietary preparation workshop to mothers in an experimental kitchen.

This study has some limitations, such as the assessment of adherence in a single moment, in such a way it is prudent to confirm the findings throughout its follow-up. Furthermore, adherence was evaluated via telephone contact, in such a way that mothers may have felt coerced into answering that they fed their children as instructed to please the researchers, in addition to education level and income being higher than those found in the general population, which compromise the generalization of the results. To the best of the authors’ knowledge, this is the first study to assess the adherence to different methods of food introduction with specific nutritional guidance and practical intervention, providing dietary workshops; hence, it is worth adding evidence to further studies on infant feeding methods.

All in all, the authors found greater adherence at 7-month-old babies to complementary feeding in a mixed approach when compared with the method for introducing food exclusively offered in a spoon and with the completely child-led approach. Thus, further studies are needed to elucidate the reasons for low adherence to these methods.
